# Preparation and Characterization of Graphene Oxide-Modified *Sapium sebiferum* Oil-Based Polyurethane Composites with Improved Thermal and Mechanical Properties

**DOI:** 10.3390/polym10020133

**Published:** 2018-01-30

**Authors:** Guiying Wu, Xiaoling Xu, Xin He, Yunjun Yan

**Affiliations:** Key Laboratory of Molecular Biophysics of the Ministry of Education, College of Life Science and Technology, Huazhong University of Science and Technology, Wuhan 430074, China; wuguiying@hust.edu.cn (G.W.); xuxiaoling@hust.edu.cn (X.X.); n785888@163.com (X.H.)

**Keywords:** graphene oxide, *Sapium sebiferum* oil, polyurethane, nano-composites

## Abstract

Bio-based polyurethane (PU) composites with superior thermal and mechanical properties have received wide attention. This is due to the recent rapid developments in the PU industry. In the work reported here, novel nano-composites with graphene oxide (GO)-modified *Sapium sebiferum* oil (SSO)-based PU has been synthesized via in situ polymerization. GO, prepared using the improved Hummers method from natural graphene (NG), and SSO-based polyol with a hydroxyl value of 211 mg KOH/g, prepared by lipase hydrolysis, were used as raw materials. The microstructures and properties of GO and the nano-composites were both characterized using Fourier transform infrared spectroscopy (FTIR), Raman spectroscopy, X-ray diffraction (XRD), transmission electron microscopy (TEM), scanning electron microscopy (SEM), thermogravimetric analysis (TGA), differential scanning calorimetry (DSC), and tensile tests. The results showed that GO with its nano-sheet structure possessed a significant number of oxygen-containing functional groups at the surface. The nano-composites containing 1 wt % GO in the PU matrix (PU1) exhibited excellent comprehensive properties. Compared with those for pure PU, the glass transition temperature (*T*_g_) and initial decomposition temperature (IDT) of the PU1 were enhanced by 14.1 and 31.8 °C, respectively. In addition, the tensile strength and Young’s modulus of the PU1 were also improved by 126% and 102%, respectively, compared to the pure PU. The significant improvement in both the thermal stability and mechanical properties for PU/GO composites was attributed to the homogeneous dispersion and good compatibility of GO with the PU matrix. The improvement in the properties upon the addition of GO may be attributable to the strong interfacial interaction between the reinforcing agent and the PU matrix.

## 1. Introduction

Polymeric nano-composites with low costs, excellent properties, and corrosion resistance derived from eco-friendly resources are extremely scarce and urgently needed in the fields of machinery fabrication, electronics, biomedicine, and the chemical industry [1,2]. In recent years, utilization of renewable resources for the synthesis of polymers has become a priority to meet the requirements of sustainable development and environment friendliness in the chemical industry [3,4].

Bio-based polyurethane (BPU) is one of the most promising polymer matrixes for composites owing to its molecular designability, structural controllability, and performance diversity [5,6]. However, polyurethane (PU) is usually made from diisocyanates and polyols originating from petroleum, which is in finite supply, and its utilization may contribute to environmental pollution.

Currently, significant attention has been focused on plant oil-based polyols, derived from soybean, sunflower, jatropha, linseed, castor, tung, palm, and *Sapium sebiferum* kernel oils, for the preparation of novel specific polyurethane formulations [7,8,9]. Compared with other plants oils, *Sapium sebiferum* oil (SSO) is one of the most economical oil sources for the preparation of *Sapium sebiferum* polyols (SSP) due to its large number of carbon-carbon double bonds and high iodine value. It is a non-food oil and can be produced on land unsuitable for farming [10]. The abundance of unsaturated bonds make the oil a desirable feedstock for the synthesis of polyols with high hydroxyl values through biochemical processes [11,12]. For this reason, using plant oil-based polyols to replace petroleum-based polyols as raw materials for the preparation of PU is a significant development, which has economic benefit and is of practical significance. However, the limited thermal stability and mechanical strength of bio-based PU materials prohibit their wide industrial application [13].

Nanomaterials with reticular, lamellar, tubular, and fibrous structures have become widely used as reinforcing agents to improve the performance of BPU composites [14,15]. Among these, graphene, a two-dimensional nanomaterial with a honeycomb lattice structure, excellent performance, and relatively low price, is an effective reinforcing agent for preparing PU composites [16,17]. Since it was discovered in 2004, graphene has received significant attention and has become a popular compound in the field of novel composites [18].

However, graphene, being neither water nor oil wettable, displays weak interactions with polymeric materials and does not disperse easily [19,20]. As a result, genuine composites with desirable properties cannot be generated. On the contrary, graphene oxide (GO), derived from graphene with a large number of oxygen-containing groups, has good wettability, surface activity, and reactivity [21]. Consequently, GO is a potential reinforcing agent for the development of high-performance composites. In previous studies, blends of GO and petroleum-based PU have been prepared [22,23,24,25]. In contrast, the modification of plant oil-based PU with GO has not been reported.

In this work, GO containing carboxylic acid and hydroxy groups at the surface has been prepared using an improved Hummers method and cavitated via ultrasonic exfoliation. Then, GO-modified SSO-based PU composites were prepared via in situ polymerization for the first time to generate novel nano-composites with improved properties. Therefore, the goals of this study were (1) to prepare and characterize GO, (2) to synthesize GO-modified SSO-based PU nano-composites, and (3) to demonstrate the reinforcement effect of GO on the nano-composites.

## 2. Materials and Methods

### 2.1. Materials

Sulfuric acid (H_2_SO_4_, 98 wt %), hydrochloric acid (HCl, 37.5 wt %), potassium permanganate (KMnO_4_), sodium nitrate (NaNO_3_), hydrogen peroxide (H_2_O_2_), and acetone were commercially obtained from Sinopharm Chemical Reagent Ltd., Co. (Shanghai, China). Nitrogen was purchased from Wuhan Xiangyun Gas Ltd., Co. (Wuhan, China). Natural graphite (NG) was got from Qingdao Furunda Graphite Ltd., Co. (Qingdao, China). Isophorone diisocyanate (IPDI) was bought from Aladdin Chemistry Ltd., Co. (Shanghai, China). *Candida rugosa* lipase was bought from Sigma-Aldrich (Shanghai, China) and used without further purification. *Sapium sebiferum* oil (SSO) was purchased from a refinery factory in Dawu County (Dawu, China). The SSP prepared from SSO was dried at 60 °C for 12 h under a vacuum to remove moisture. Other materials were of analytical grade and used without further treatment.

### 2.2. Synthesis Methods

#### 2.2.1. Preparation of GO

**Scheme 1:**
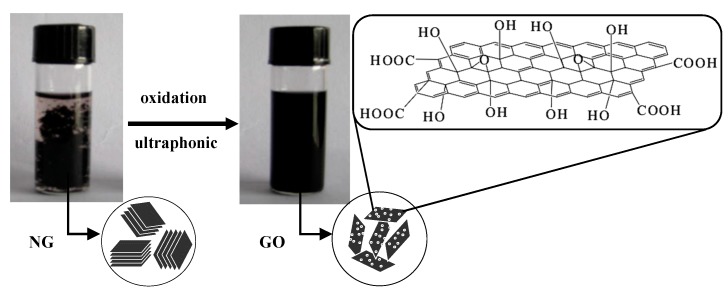
Synthesis of graphene oxide (GO).

Exfoliated GO was synthesized by following the improved Hummers method [26,27], and treated to ultrasonic exposure. First, 1 g of NG flakes and 0.5 g of NaNO_3_ were dissolved in 75 mL of 98 wt % H_2_SO_4_ under magnetic stirring at 400 rpm in an ice-water bath for 30 min. 3 g of KMnO_4_ was then added gently into the solution, whereby the temperature of the solution did not exceed 20 °C. After the accomplishment of the addition, the reaction mixture was stirred continuously for 2 h at 35 °C. Second, 200 mL of deionized water was slowly added to dilute the viscous solution, which was then stirred at 98 °C for a further 12 h. After the dilution, 3 mL of H_2_O_2_ (30 wt %) was added and allowed to react for 0.5 h. Then, the brown precipitation (GO) was purified and washed with moderate HCl (5%) and deionized water by repeated centrifugation until the solution became neutral. Thereafter, the product was dispersed in deionized water and ultrasonicated for 30 min until a homogeneous solution was obtained (see Scheme 1). The unexfoliated GO was then removed using centrifugation at 12,000 rpm for 5 min, and the as-prepared GO was dried at 40 °C at reduced pressure for later use.

#### 2.2.2. Synthesis of PU and PU/GO Composites

The SSP was synthesized based on SSO in a continuous process of epoxidation-hydroxylation and enzyme-catalyzed methods, as detailed in our previous work [28]. Simply, a two-step reaction sequence of epoxidation with peracetic acid, followed by hydroxylation with methanol, leading to a general process and avoiding unnecessary intermediate steps. Then, the aforementioned obtained ester glycerides were partially catalyzed in the presence of *Candida rugosa* lipase without any surfactant or organic solvent. The generated polyol product showed a hydroxyl value of 211 mg KOH/g, and the sample was designated as polyol-211.

**Scheme 2:**
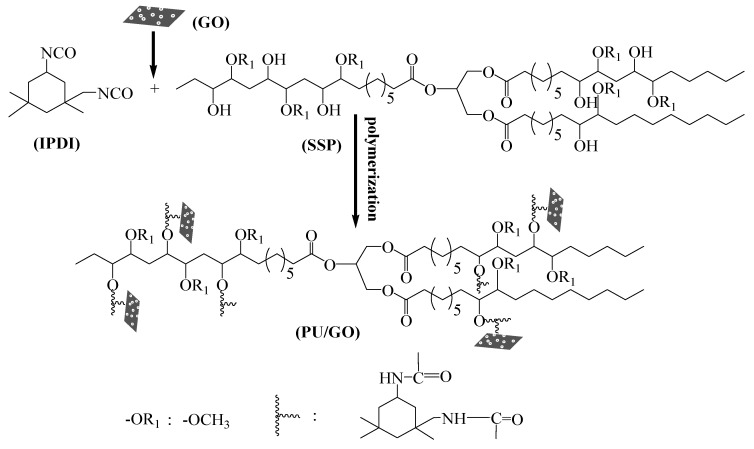
Synthesis process of polyurethane (PU)/GO composites.

SSO-based PU was further synthesized by the reaction of IPDI and polyol-211 according to a previously reported method [28,29,30]. The PU/GO composites were prepared via the reaction of IPDI and polyol-211 with different contents of GO at 0, 0.5%, 1%, and 1.5%, named as PU0, PU0.5, PU1, and PU1.5, respectively, as shown in Scheme 2 [29,30]. GO and 20 mL of acetone were mixed sufficiently using sonication for 30 min. The mixture and IPDI were allowed to react in a water-cooled condenser with a magnetic stirring at 80 °C for 2 h under a nitrogen atmosphere. After that, the solution was cooled to 60 °C, and polyol-211 was added (1.1:1 mole ratio of –NCO to –OH groups) to react for 2 h. The obtained product was degassed under vacuum for 10 min, poured onto polytetrafluoroethylene (PTFE) molds while hot, and then heated to solidify in an oven at 60 °C for 8 h.

### 2.3. Testing and Measurement

#### 2.3.1. Fourier Transform Infrared Spectroscopy (FITR)

A Fourier transform infrared spectrometer (Vertex 70 FTIR, Bruker Company, Karlsruhe, Germany) was used to examine the significant absorption spectra due to the stretching, bending, and vibration of the various types of chemical bonding present in NG, GO, PU, and PU composites at room temperature in the range of 4000–400 cm^−1^ at a resolution of 4 cm^−1^.

#### 2.3.2. Raman

A laser Raman spectrometer (Vertex 70 Raman, Bruker Company, Karlsruhe, Germany) was employed to analyze the structures of NG and GO by a He-Ne (632.8 nm) laser using a Labram 300 system.

#### 2.3.3. X-ray Diffraction (XRD)

An X-ray diffractometer (Empyrean XRD, PANalytical B.V., Almelo, The Netherlands) was used to analyze the crystallization property of NG and GO. The XRD patterns were recorded with a Cu *K*α radiation (40 kV and 40 mA) at an incident wavelength of 0.15418 nm in a Ni filter, with a 2θ range of 5–50°, a step size of 0.02°, and a testing time of 0.1 s.

#### 2.3.4. Morphology Analysis

Transmission electron microscopy (TEM) (Tecnai G2 20 FEI instrument, Eindhoven, Holland, The Netherlands) was designed to determine the presence of the exfoliated GO. The samples were diluted in ethanol and well dispersed for 15 min by sonification. A drop of the sample solution was then placed on a copper grid using a micropipette and dried before TEM observation.

The morphology of pure PU and its composites were both observed with scanning electron microscopy (Nova Nano SEM 450, FEI Company, Eindhoven, The Netherlands) at an acceleration voltage of 10 kV and a spot size of 10 nm. All specimens were freeze-fractured in liquid nitrogen and coated with gold before SEM observation.

#### 2.3.5. Thermal Stability Analysis

The thermal properties of the PU and PU/GO composites were analyzed with thermogravimetric analysis (Pyris1 TGA, Perkin-Elmer Instruments, Boston, MA, USA), from 30 to 600 °C at a heating rate of 10 °C·min^−1^ under a nitrogen atmosphere. The weight of the measured samples was about 5 mg.

The glass transition temperatures (*T*_g_) of the PU and PU/GO composites were tested by differential scanning calorimetry (Diamond DSC, Perkin-Elmer Instruments, Boston, MA, USA) from 0 to 120 °C with a heating rate of 10 °C·min^−1^ under a nitrogen flow. Samples of approximately 5 mg were placed in an aluminum pan for each run.

#### 2.3.6. Mechanical Testing

The mechanical properties of the PU and PU/GO composites (100 mm × 10 mm × 4 mm) were examined on a CMT4104 universal testing machine (Shenzhen SANS Testing Machine Ltd., Co., Shenzhen, China) at a speed of 50 mm·min^−1^. Every specimen was measured six times, and the average value was calculated.

## 3. Results and Discussion

### 3.1. Structural Characterization of GO

#### 3.1.1. FTIR Analysis of GO

**Figure 1:**
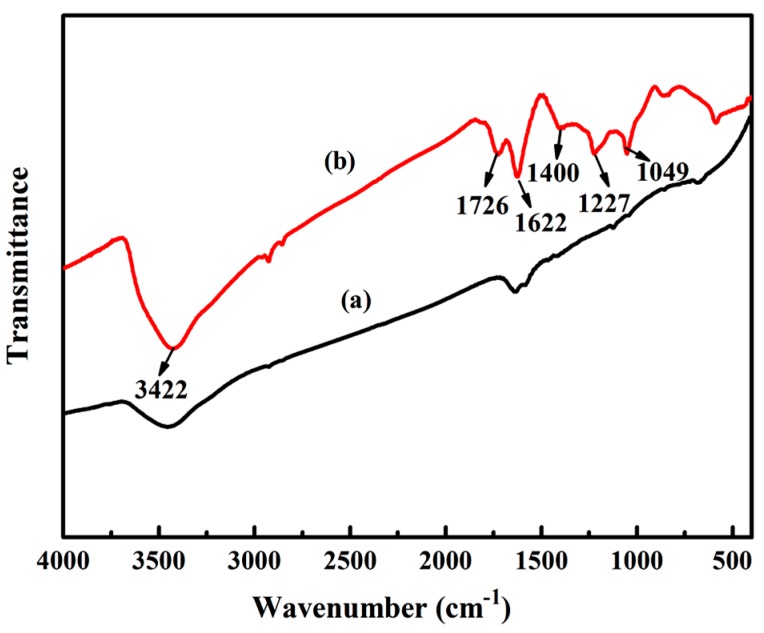
Fourier transform infrared spectroscopy (FITR) spectra of natural graphene (NG) (**a**) and GO (**b**).

The FTIR spectra for NG and GO are shown in Figure 1a,b, respectively. The band at 3422 cm^−1^ is attributed to the presence of O–H groups on the surface of NG and is recognized as the tight binding of moisture restored in NG. The weak absorption at 1622 cm^−1^ is the characteristic of the C=C stretching vibration between the layers of NG. For the GO spectrum(Figure 1b), the peaks at 3422, 1726, 1622, 1400, 1227 and 1049 cm^−1^ are assigned to O–H stretching, C=O stretching, C=C stretching, bending vibration of O–H in carboxylic group, C–OH stretching, and C–O stretching [31,32]. This indicated that some of the –OH had been enriched on the GO surface [33]. The above results confirm the successful oxidation of NG, and GO could be successfully produced by using the improved Hummers method.

#### 3.1.2. Raman Analysis of GO

**Figure 2:**
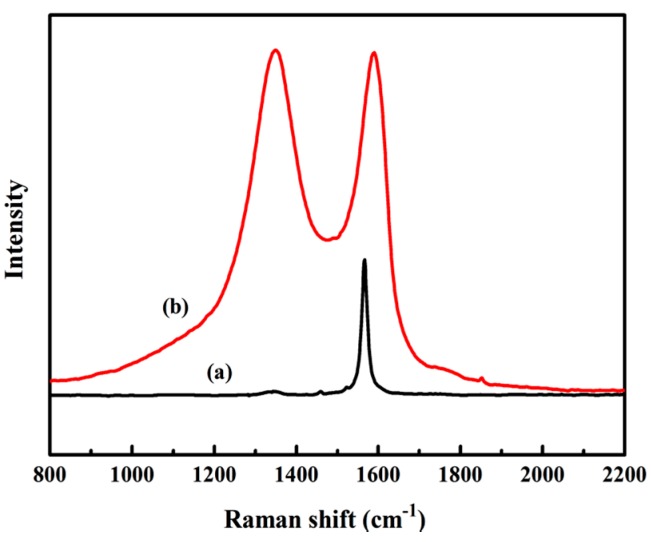
Raman spectra of NG (**a**) and GO (**b**).

Figure 2 shows the Raman spectra of NG (a) and GO (b). GO exhibits two intensity peaks, namely, the D-bond around 1348 cm^−1^ and the G-bond around 1590 cm^−1^. In comparison, NG exhibits a weak D peak and an apparent G peak. The D-bond is associated with the in-plane bond-stretching motion of the C sp^2^ atom pairs, which is attributed to the defects and disorders in the hexagonal graphitic layers. The G-band is concerned with the breathing modes of rings [34]. The large value of IG/ID (intensity of G/D peak) indicates that NG has a highly ordered architecture. For GO, the value of IG/ID decreases obviously, illustrating the irregularity structure of GO [35]. This is attributable to the structural destruction of GO, with a large number of oxygen-containing groups introduced into the graphite layers after oxidation and ultrasonic stripping treatment of NG. This hints that the random layer structure of GO may display a better compatibility in the PU matrix.

### 3.2. XRD Analysis of GO

**Figure 3:**
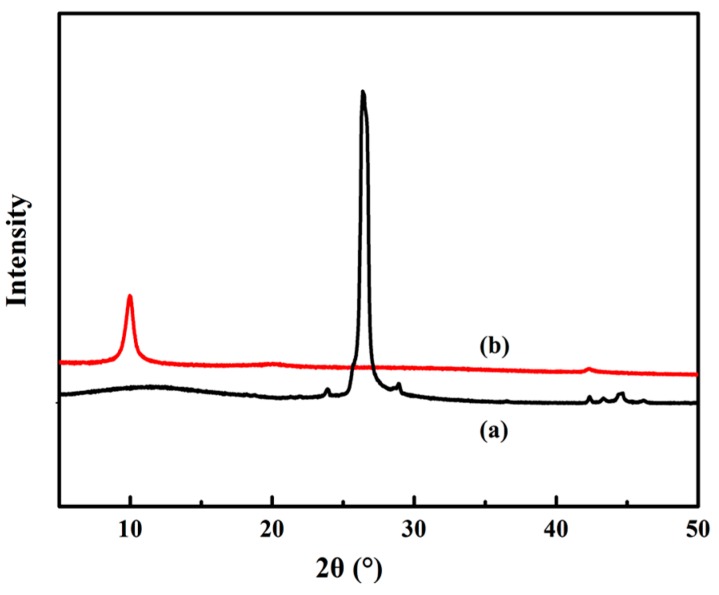
X-ray diffraction (XRD) patterns of NG (**a**) and GO (**b**).

The magnitude and location of peaks in the XRD curves (Figure 3) provides very useful information for NG (a) and GO (b). NG shows a sharp and high intensity reflection peak at 2θ of 26°, illustrating that the microcrystalline laminar contained in NG is tidily arranged. The weak intensity and sharp reflection peak of GO at 2θ of 10° is due to the introduction of functional groups, such as hydroxyls and carboxyls, during the oxidation and ultrasonic treatment. The results indicate that oxygen-containing functional groups were grafted in the layer of NG, the crystalline structure of NG was destroyed and the laminar spacing was increased [36]. This infers that the oxidation and ultrasonic treatment of NG plays effective roles in the preparation of GO. This kind of structure leads to an improvement in the compatibility and comprehensive performance of the composites.

### 3.3. TEM Analysis of GO

**Figure 4:**
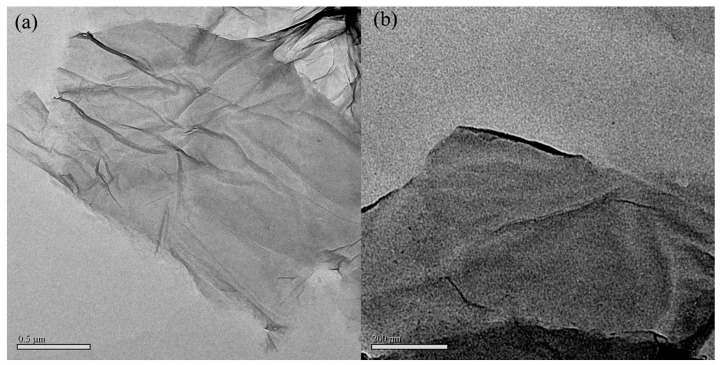
The transmission electron microscopy (TEM) images of GO at (**a**) in 0.5 μm and (**b**) in 200 nm magnifications.

Figure 4 shows the TEM images of the morphology of GO at 0.5 μm (a) and 200 nm (b) magnifications. GO displays a transparent and pleated sheet structure with nanoscale thickness. The pleated structure of GO possesses a lower energy state than a stretchy structure, which ensures the structural stability of GO [37]. In addition, the transparency indicates that the exfoliated GO exists in a single layer or a few layers. Furthermore, owing to the oxidation of NG, oxygen-containing functional groups were introduced into the surface of GO, and the orderly structure of the layers might be broken, which would weaken the intra-molecular interactions and enhance the dispersion stability.

### 3.4. Structural Characterization of PU/GO Composites

**Figure 5:**
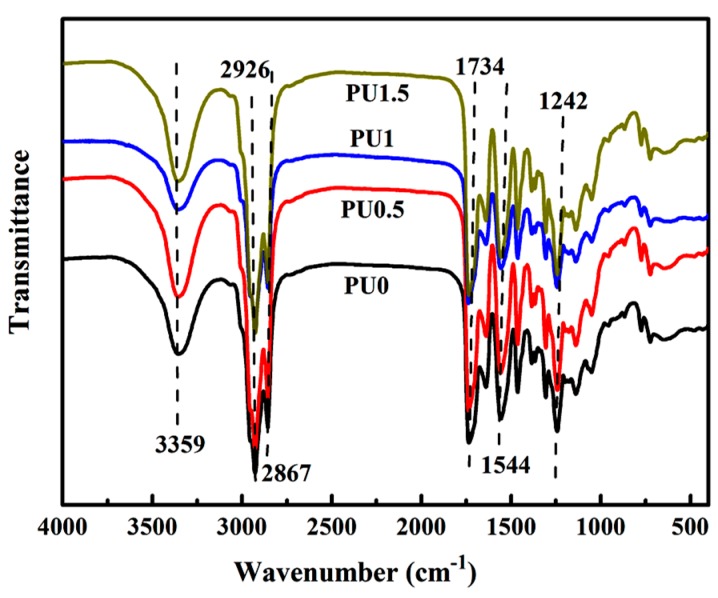
Fourier transform infrared spectroscopy (FTIR) spectra of PU0 and PU/GO composites.

The FTIR spectra of PU and PU/GO with different proportions of GO are presented in Figure 5. The characteristic absorption of –OH groups in the PU and PU/GO composites is obvious at 3359 cm^−1^, which is shifted slightly to lower wavenumbers direction in comparison with that in GO (Figure 1b). Unexpectedly, although isocyanate groups were overdosed in the original formulation, no –NCO peak was observed at 2270 cm^−1^. One possible reason was that not all the –OH groups were accessible to react with –NCO groups due to the existence of molecular steric hindrance. The other probable cause was that the –NCO groups reacted with –OH derived from the moisture in the air [38]. In addition, the typical characteristic infrared absorption peak of PU appeared. The broad bands at 3359 and 1726 cm^−1^ were attributed to urethane N–H stretching and C=O stretching. The two peaks at 1544 and 1232 cm^−1^ corresponded to the N–H in-plane and C–N bond stretching, respectively [38]. The stretching vibration bands at 2926 and 2867 cm^−1^ were assigned to methyl (–CH_3_) and methylene (–CH_2_–) in the composites. The characteristic peaks of C–O at 1242 cm^−1^ were the vibration bands of the ester bond. In general, the obtained results demonstrated that PU was successfully synthesized, with no significant difference between PU and PU/GO. In other words, the addition of GO did not introduced any new functional groups into the PU matrix.

### 3.5. Microstructure of Pure PU and PU/GO Composites

**Figure 6:**
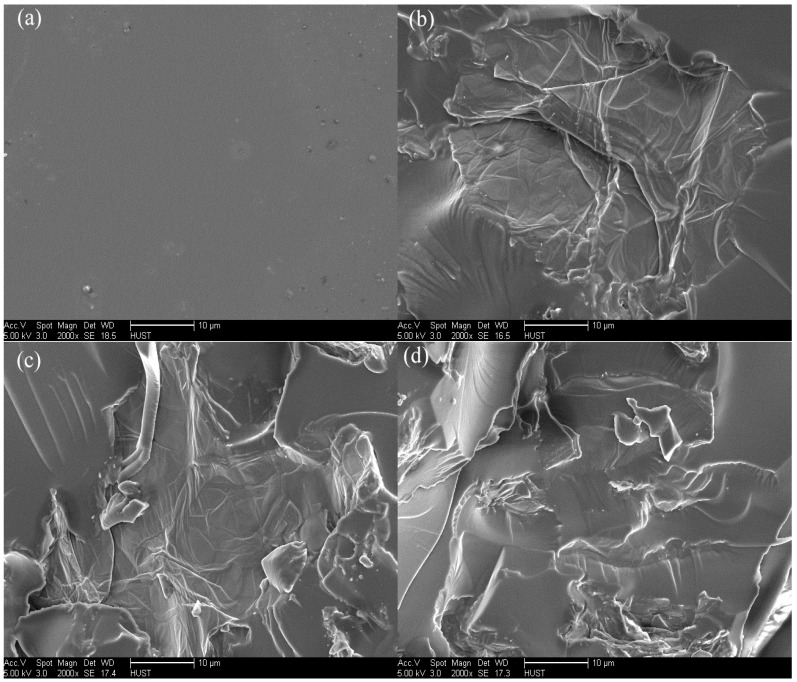
Scanning electron microscopy (SEM) images of PU0 (**a**), PU0.5 (**b**), PU1 (**c**), and PU1.5 (**d**) at a 10 μm magnification.

Figure 6 shows the SEM images of the dispersion and interactions of GO in the PU matrix. As shown, the fractured surface of PU0 is smooth(Figure 6a), while the laminar folds in the images represent GO nano-sheets in the PU matrix and rough fractured surfaces displays on the PU/GO composites. A non-separated structure appears on the fracture section of the composite with GO for different mass fractions, which demonstrates that the exfoliated GO flakes can be adequately embedded into the PU matrix. When the GO is at 1 wt % loading (Figure 6c), the fractured surface of the composite fills with GO nano-sheets and generates a tight binding and homogeneous dispersion, suggesting that good compatibility and strong interactions exist between GO and the PU matrix. A slight agglomeration and a cumulative folding surface (Figure 6d) appears at a GO loading of 1.5 wt %, resulting in a rather poor distribution of GO in the matrix. This is probably attributed to the strong hydrophilicity and high surface free energy of GO nano-sheets when the loading is further increased. Hence, GO partially grafting on the PU matrix can provide a homogeneous dispersion, which effectively improves the overall properties of the PU/GO composites.

### 3.6. Thermal Properties of Pure PU and PU/GO Composites

**Figure 7:**
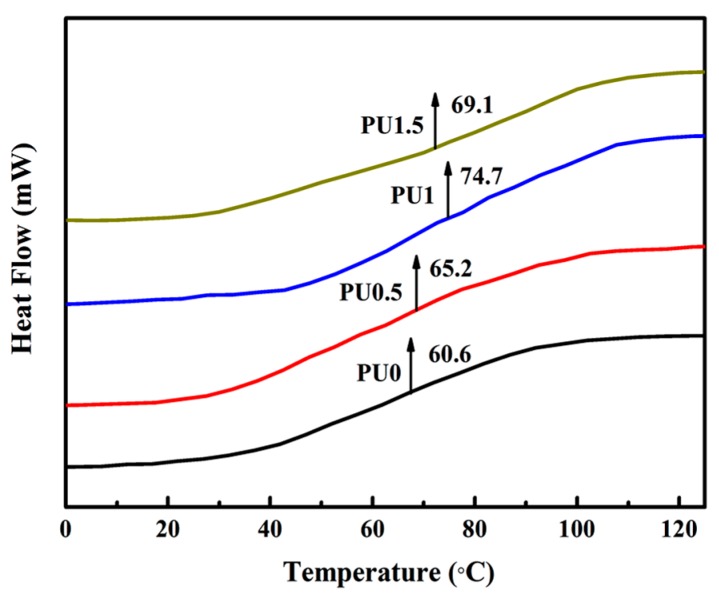
Differential scanning calorimetry (DSC) curves of PU0 and PU/GO composites.

DSC experiments provided the glass transition temperature (*T*_g_) for the pure PU and PU/GO composites. As seen in Figure 7, the *T*_g_ values of PU0, PU0.5, PU1 and PU1.5 are 60.6, 65.2, 74.7 and 69.1 °C, respectively. The *T*_g_ of all PU/GO composites are higher than that of the pure PU, suggesting that GO has a significantly positive effect on the crystallization of the PU, resulting in an increase in *T*_g_. As reported in the literature, *T*_g_ represents the mobility of polymer chains and network in the matrix at the molecular level [39]. The increase in the value of *T*_g_ indicates a movement decrease for molecules in the matrix.

In this experiment, the well-distributed GO nanomaterial in the matrix acts as a reinforcing agent, which enhances the rigidity of the matrix and limits the free movement of the molecular chain. PU1 had a maximal value of 74.7 °C. The value of *T*_g_ for PU1.5 decreased slightly compared with that of PU1, which was due to the micro-phase separation of the PU/GO composites, resulting in part of the agglomeration owing to the overloading of GO in the matrix. This was also confirmed by the SEM images. Therefore, the well-distributed GO nano-sheets in the PU matrix can efficiently increase the value of *T*_g_.

**Figure 8:**
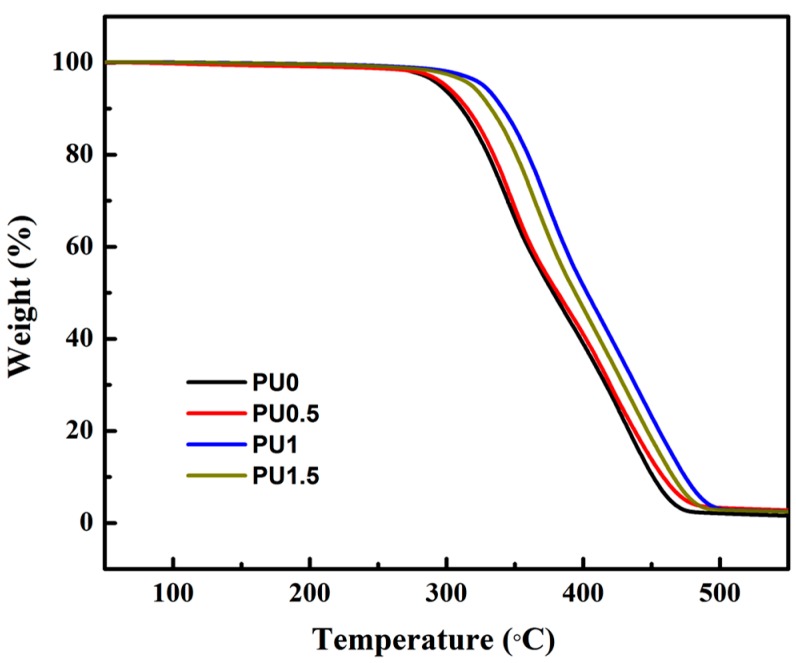
Thermogravimetric analysis (TGA) curves of PU0 and PU/GO composites.

**Figure 9:**
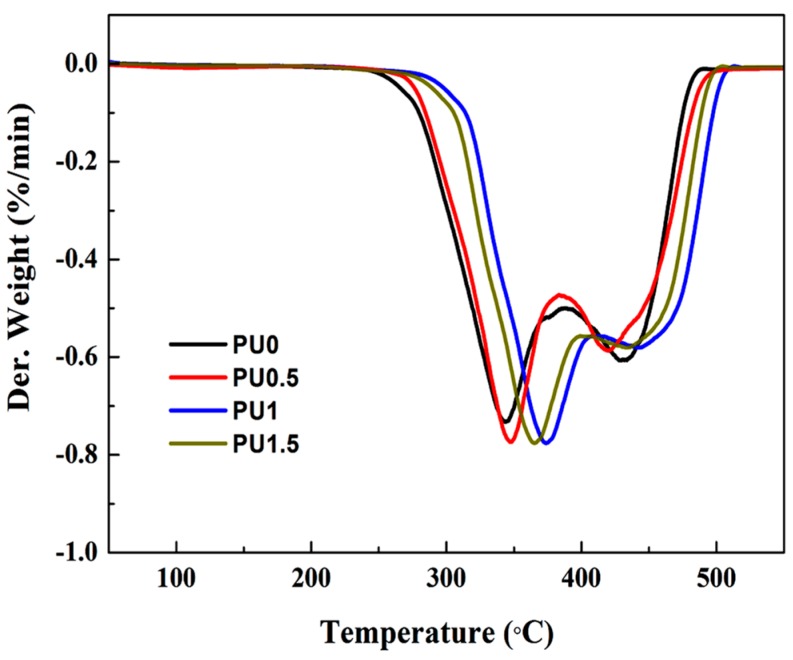
Differential thermal gravity (DTG) curves of PU0 and PU/GO composites.

Figure 8 and Figure 9 show the TGA and DTG curves of the PU and PU/GO composites, respectively. The values of initial decomposition temperature at 5% weight loss (IDT), temperature for the maximum rate of degradation (*T*_max_) and residues are listed in Table 1. As seen in the TGA curves, the same trend of weight loss displays for all samples. A small weight loss at 100 °C was due to the decomposition of bound moisture, low molecules organic solvents and impurity. The other two obvious weight loss processes are the decomposition of the soft segment at the range of 340–380 °C and the decomposition of the hard segment at the range of 420–450 °C. Thermal degradations of the pure PU and PU/GO composites take place at two stages, corresponding to the thermodynamic incompatibility of the two segments of the PU matrix [40].

The IDT of PU0 is 294.3 °C, which is lower than that of PU/GO composites (Table 1). The *T*_max_ for both of the two stages of the PU/GO composites is also much higher than that of PU0. As expected, the incorporation of 1 wt % GO in the PU matrix displays the best thermal stability of the composites. PU1 displays the highest IDT and two step of *T*_max_, which delayed by 31.8, 33.4 (1st) and 12.9 °C (2nd) at the side of PU0. It is attributable to the homogeneous dispersion and stronger interfacial interactions of GO in the PU matrix. The residues of the PU and PU/GO composites at 550 °C are about 2 g due to the carbonization. These results suggest that an appropriate loading of GO in the PU matrix can enhance the thermal stability of PU/GO composites. It can be ascribed to the numerous oxygenated active groups randomly distributed on the surface of GO, which play effective roles in undergoing chemical reactions and intermolecular interactions with the matrix. As a result, the networks were formed that limit the movement of the molecular chain of PU. Alternatively, the overloading nano-sheets of GO can generate an irreversible aggregation in some way, which decreases the thermal stability of the composites.

### 3.7. Mechanical Properties of Pure PU and PU/GO Composites

The mechanical properties of PU/GO with different contents of GO were investigated by tensile testing. The tensile strength, elongation at break and Young’s modulus are listed in Table 2. It shows that the tensile strength and Young’s modulus of PU0 are lower than those of the PU/GO composites, while a slight decrease occurs for the elongation at break of the PU/GO composites. PU1 has the highest tensile strength and Young’s modulus among the composites, and they are 28.3 and 45.1 MPa, which were, respectively, increased by 126% and 102% in comparison with PU0. Further addition of GO (1.5 wt %) into the PU matrix causes a decrease in tensile strength and Young’s modulus, which is presumably due to the partial agglomeration phenomenon of GO. It is demonstrated that it was the construction of nano-sheets of GO that are responsible for the enhanced effect. The probable mechanism is that large numbers of oxygen-containing groups are exposed on the GO surface, leading to strong covalent bonding forces and interfacial interactions between GO and PU, which can facilitate the generation of a cross-linked polymer network [17]. However, the elongation at break of the composites decreases with increasing GO content. The possible cause is the high cross-linkage between the GO and PU matrix, which reduces the toughness and malleability of the composite. These results are also in accordance with the results of *T*_g_ and *T*_max_. Therefore, the addition of a proper amount of GO into the PU matrix can significantly improve its *T*_g_, thermal stability and mechanical properties.

## 4. Conclusions

In this work, GO and oil-based PU/GO nano-composites were synthesized and characterized. The FTIR, XRD, TEM, and Raman spectroscopy results showed that GO had been successfully prepared using the improved Hummers method and partly introduced into the PU matrix via in situ polymerization. After oxidation and ultrasonic treatment, the amount of oxygen functional groups on the GO surface was greatly increased. GO exhibited a nano-sheet structure and was used as a reinforcing agent for PU improvement. The structure-property relationships with different contents of GO were illustrated. SEM analysis shows that the addition of 1 wt % GO displays the best dispersion inside the PU matrix and is the most effective to improve the thermal and mechanical properties of the PU/GO composite. The IDT and *T*_g_ of PU1 compared with PU0 enhances at 31.8 and 14.1 °C, respectively. In the meantime, the tensile strength and Young’s modulus of PU1 are also enhanced by 126% and 102%. The marked improvement of thermal and mechanical properties of the PU/GO composites is due to the good compatibility and the presence of strong interfacial interaction between GO and PU matrix. Moreover, the novel oil-based PU/GO composites in this study may have promising and comprehensive applications in the fields of machinery, electronics, biomedicine, and the chemical industry.

## Figures and Tables

**Table 1:** Dynamic mechanical analysis (TGA) and differential thermal gravity (DTG) results for PU0 and PU/GO composites.

Sample	IDT (°C)	*T*_max_		Residue at 550 °C (%)
		1st Step (°C)	2nd Step (°C)	
PU0	294.3	341.8	429.7	1.8
PU0.5	297.6	347.3	419.2	2.1
PU1	326.1	375.2	442.6	2.2
PU1.5	315.2	365.1	436.8	2.3

**Table 2:** Mechanical properties of PU0 and PU/GO composites.

	Tensile strength (MPa)	Elongation at break (%)	Young’s modulus (MPa)
PU0	12.5 ± 0.6	168.3 ± 12.3	22.3 ± 1.4
PU0.5	19.2 ± 0.8	152.2 ± 12.5	28.4 ± 1.5
PU1	28.3 ± 1.9	143.5 ± 11.4	45.1 ± 4.1
PU1.5	24.7 ± 1.6	134.6 ± 10.8	35.3 ± 3.2
